# PacBio Iso-Seq Improves the Rainbow Trout Genome Annotation and Identifies Alternative Splicing Associated With Economically Important Phenotypes

**DOI:** 10.3389/fgene.2021.683408

**Published:** 2021-07-15

**Authors:** Ali Ali, Gary H. Thorgaard, Mohamed Salem

**Affiliations:** ^1^Department of Animal and Avian Sciences, University of Maryland, College Park, College Park, MD, United States; ^2^School of Biological Sciences and Center for Reproductive Biology, Washington State University, Pullman, WA, United States

**Keywords:** rainbow trout, transcriptome, PacBio, Iso-Seq, long reads, alternative splicing, alternative polyadenylation, exon usage

## Abstract

Rainbow trout is an important model organism that has received concerted international efforts to study the transcriptome. For this purpose, short-read sequencing has been primarily used over the past decade. However, these sequences are too short of resolving the transcriptome complexity. This study reported a first full-length transcriptome assembly of the rainbow trout using single-molecule long-read isoform sequencing (Iso-Seq). Extensive computational approaches were used to refine and validate the reconstructed transcriptome. The study identified 10,640 high-confidence transcripts not previously annotated, in addition to 1,479 isoforms not mapped to the current Swanson reference genome. Most of the identified lncRNAs were non-coding variants of coding transcripts. The majority of genes had multiple transcript isoforms (average ∼3 isoforms/locus). Intron retention (IR) and exon skipping (ES) accounted for 56% of alternative splicing (AS) events. Iso-Seq improved the reference genome annotation, which allowed identification of characteristic AS associated with fish growth, muscle accretion, disease resistance, stress response, and fish migration. For instance, an ES in *GVIN1* gene existed in fish susceptible to bacterial cold-water disease (BCWD). Besides, under five stress conditions, there was a commonly regulated exon in prolyl 4-hydroxylase subunit alpha-2 (*P4HA2*) gene. The reconstructed gene models and their posttranscriptional processing in rainbow trout provide invaluable resources that could be further used for future genetics and genomics studies. Additionally, the study identified characteristic transcription events associated with economically important phenotypes, which could be applied in selective breeding.

## Introduction

Rainbow trout is one of the most important fish species that significantly contributes to the aquaculture industry of the United States and has been extensively used as a model organism for biomedical research. International efforts have been ongoing over the years to develop genomic and transcriptomic resources for this species (Salem et al., 2010, 2015; Ali et al., 2014; Berthelot et al., 2014; Al-Tobasei et al., 2016). The Sanger sequencing approach has been considered as the gold standard for sequencing full-length (FL) cDNA clones and genome annotation (Denoeud et al., 2008). This approach was previously used with the 454 pyrosequencing technology to assemble the rainbow trout transcriptome yielding transcripts with an average length below 1 kb (Salem et al., 2010). Sanger sequencing fell behind when cheaper short-read technologies came out to refine the rainbow trout transcriptome (Fox et al., 2014; Salem et al., 2015). The rainbow trout genome assembly (Berthelot et al., 2014), released in 2014, failed to completely cover and adequately anchor a high percentage of genes to chromosomes. More recently, the genome assembly and gene spaces were further refined (Pearse et al., 2020). Despite the accumulation of massive short-read data over recent years, the lack of FL transcripts has been a significant limitation to define alternatively spliced and polyadenylated transcripts leading to incorrect or incomplete gene annotations (Au et al., 2013; Abdel-Ghany et al., 2016). Transcript reconstruction methods for short reads achieved good precision at the exon level, but the accuracy was low to assemble complete transcripts even in species with simple transcript structures (Steijger et al., 2013). Short reads can accurately identify splice sites but are limited to infer splice site usage and discover transcript isoforms (Steijger et al., 2013; Wang et al., 2016).

Alternative splicing (AS) is a predominant phenomenon in eukaryotic genomes that increases the repertoire of proteins without increasing the number of genes (reviewed in Kornblihtt et al., 2013). In humans, ∼95% of the multi-exonic genes undergo AS (Pan et al., 2008; Barash et al., 2010) and, thus, facilitate the evolution of complex functional transcriptomes capable of regulating various molecular, cellular, and developmental processes (Kalsotra and Cooper, 2011; Seo et al., 2013). In *Drosophila*, the *DSCAM* gene alternatively splices to generate more than ∼38,000 isoforms equivalent to ∼2.5 the number of genes in the fly (Schmucker et al., 2000). The biological functions of multiple isoforms are poorly explored; however, some studies provided evidence for the mechanistic regulatory role of AS. For example, the *Bcl-x* gene of the fruit fly generates two transcript isoforms coding for antagonistic proteins where one isoform activates apoptosis and the other inhibits it (Li et al., 2004). In humans, AS due to skipping exon 7 of the *SMN* (survival motor neuron) has been demonstrated to directly correlate with spinal muscular atrophy (Zhou et al., 2008). Conversely, the inclusion of exon 10 in tau transcript due to abnormal splicing has been implicated in tauopathies (Zhou et al., 2008). Clinical strategies are underway to target aberrant AS associated with human diseases (Zhou et al., 2008, 2012).

In addition to AS, recent RNA sequencing studies showed that alternative cleavage and polyadenylation contribute to transcriptome complexity and diversity in higher organisms (Wu et al., 2011; Sherstnev et al., 2012). Although RNA-Seq provides massive depth and understanding of the transcriptome, RNA-Seq protocols are behind in resolving transcript termini (Steijger et al., 2013). Therefore, other methods for sequencing 3′ and 5′ ends were adopted to retrieve requisite information. Cap analysis for gene expression (CAGE) sequencing has been used to annotate transcription start sites (TSSs) (Main et al., 2013; Boley et al., 2014), whereas deep 3′-sequencing (3′-seq) was used to define transcript termini and reveal unexpected alternative polyadenylation (APA) patterns (reviewed in Miura et al., 2014). In the human complex transcriptome, 54% of genes have multiple TSSs (Tyner et al., 2017). Precise promoter annotation will help to investigate the 5′ untranslated region (UTR) differential usage and the functional impact of genetic variation on gene expression. For instance, a regulatory single nucleotide polymorphism creates a new TSS causing thalassemia (De Gobbi et al., 2006). 3′ UTRs are the major mediators for posttranscriptional regulatory mechanisms, and therefore, gain or loss of regulatory elements such as microRNA binding sites, due to APA, can affect transcript stability and translational efficiency (reviewed in Miura et al., 2014). Although specialized methods in resolving transcript termini are available, none of the technologies mentioned above provides insights into the complete transcript structure.

The single-molecule real-time (SMRT) Iso-Seq of Pacific Biosciences (PacBio) allows a comprehensive analysis of the transcriptome. Unlike short-read RNA-Seq, Iso-Seq can capture full-length sequences, thereby improves gene annotation and accurately identifies transcript isoforms and gene fusions (Nudelman et al., 2018; Feng S. et al., 2019; Tian et al., 2019). Besides, long-read sequencing provides clear evidence for posttranscriptional processes such as APA and splicing events (Treutlein et al., 2014; Abdel-Ghany et al., 2016). Thus far, long-read sequencing has not widely been used in fish, with few reports in *Danio rerio* (Nudelman et al., 2018), *Lateolabrax maculatus* (Tian et al., 2019), *Misgurnus anguillicaudatus* (Yi et al., 2018), *Gymnocypris selincuoensis* (Feng X. et al., 2019), and *Salmo salar* (Ramberg et al., 2021). Conducting similar analyses in other species will contribute to understanding AS and the regulatory roles of APA and reveal the evolutionary conservation of splice isoforms (Abdel-Ghany et al., 2016).

In this study, PacBio long-read transcriptome sequencing was applied to improve the rainbow trout transcriptome annotation and yield a catalog of high-confidence transcript isoforms. We sequenced 14 tissues from three doubled haploid YY males from the Swanson River clonal line to achieve high coverage of transcript isoforms. In parallel, short-read RNA-Seq datasets were used to validate splice sites and AS events. The study findings revealed that intron retention (IR) is the most frequent AS event. The corrected PacBio transcriptome has been used to study the plasticity in exon usage in association with several physiological conditions of the fish. This study demonstrated the utility of PacBio Iso-Seq platform to characterize FL cDNA sequences and identify novel genes/isoforms, improving genome annotation and extending our knowledge/understanding of the rainbow trout transcriptome beyond the currently available resources.

## Results and Discussion

### Iso-Seq Analysis Pipeline

**FIGURE 1:**
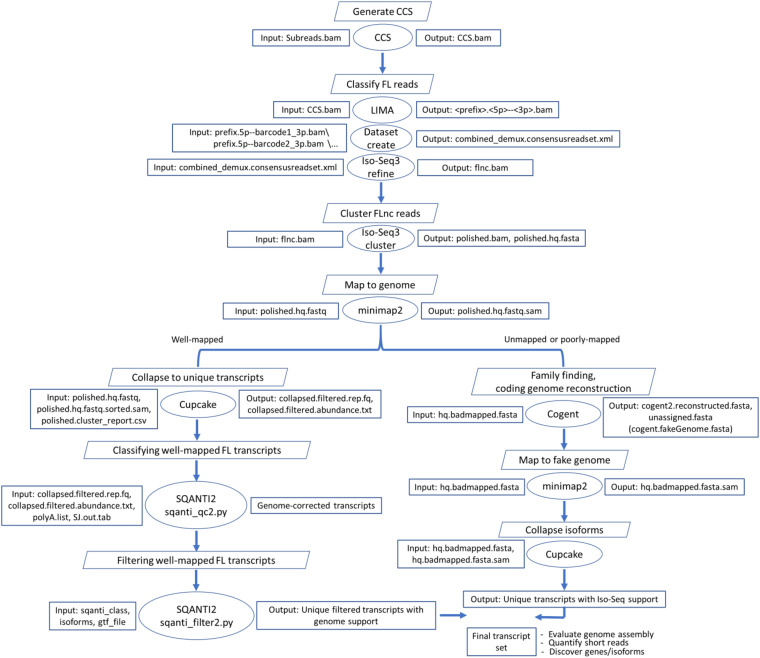
Bioinformatics pipeline to reconstruct the rainbow trout transcriptome from the Iso-Seq dataset. CCS.bam file contains circular consensus sequence (CCS) reads, flnc.bam contains full-length non-chimeric (FLnc) reads, SJ.out.tab contains high confidence collapsed splice junctions (tab-delimited format), and polyA.list contains a list of polyA motifs to find upstream of the 3′ end site. “hq” stands for high quality.

Large-scale sequencing is essential for gene discovery and genome annotation; however, the sequencing depth, sequence completeness, and cost are the main limitations of sequencing technologies (Wang et al., 2016). EST sequences and 454 pyrosequencing were previously used to assemble the trout transcriptome (Salem et al., 2010). Sanger sequencing is relatively expensive and generated sequences shorter than 1 kb. The 454 pyrosequencing produced ∼1.3 million reads (344 bp long on average) shorter than the EST sequences (Salem et al., 2010). Recently, Illumina short-read sequencing provided high sequencing depth, which assisted in refining the transcriptome (Berthelot et al., 2014; Salem et al., 2015; Pearse et al., 2020) and providing insights into transcriptional networks (Ali et al., 2018; Paneru et al., 2018) and gene structure (Berthelot et al., 2014; Pearse et al., 2020). However, short-read RNA-Seq breaks the continuity of the transcript and, therefore, fails to reconstruct the actually expressed transcripts and impairs our understanding of the functional aspects of isoform diversity (Steijger et al., 2013; Tilgner et al., 2014). More recently, PacBio Iso-Seq has been extensively used to identify FL transcripts and improve genome annotation (Abdel-Ghany et al., 2016; Wang et al., 2016; Nudelman et al., 2018; Feng S. et al., 2019; Feng X. et al., 2019; Tian et al., 2019). To characterize the rainbow trout transcriptome using Iso-Seq, RNA samples were isolated from 14 tissues in addition to a pooled RNA sample from fertilized eggs at different embryonic developmental stages. Tissues were collected from three doubled haploid fish to reduce heterozygosity but maintain tissue specificity. Twenty samples from two fish were barcoded and sequenced on four SMRT cells. To obtain a higher yield per tissue, 15 samples from one more fish were sequenced using SMRT cell per tissue. To reconstruct a high-confidence FL transcriptome, the ToFU pipeline (Isoseq3 v3.2.2) (Gordon et al., 2015) was used as illustrated in Figure 1. PacBio sequencing yielded a total of 6,776,786 reads of inserts (RoIs). Circular consensus sequencing (CCS) reads were generated and classified into 5,411,377 (79.9%) full-length non-chimeric (FLnc) reads of length ranges from 50 up to 25,831 bp (avg. = 2.3 kb). FLnc reads were defined as sequences having 5′ and 3′ barcoded primers and the poly(A) tail. Reads lacking any of these requirements were classified as non-full length (nFL) and were excluded from the analyses. In sea bass (*Lateolabrax maculatus*), 42.5% of the reads were classified as FL (Tian et al., 2019). The high percentage of the FLnc reads (79.9%) indicates high integrity of the trout RNAs used in the current Iso-Seq study.

The iterative clustering for error correction (ICE) algorithm was used in the Iso-Seq pipeline to obtain clusters of FL reads and then compute FL consensus isoform sequences (Figure 1). High-quality consensus sequences (452,955 FLnc) were mapped to the rainbow trout genome (NCBI Omyk_1.0) (Pearse et al., 2020) using the minimap2 alignment tool. A total of 451,178 reads (99.61%) were mapped to the reference genome, suggesting that the error rate of PacBio raw data, if any, was successfully corrected by the ICE as previously reported (Gordon et al., 2015). The percentage of unmapped reads (0.4%) was lower than that (3.6%) reported for zebrafish (Nudelman et al., 2018). The mapped reads were collapsed using the Cupcake tool, yielding 108,501 non-redundant isoforms (average length ∼2.8 kb) exhibiting alignment identity ≥ 0.95 and alignment coverage ≥ 0.99. To avoid truncated transcripts, incomplete retrotranscription reads differing only in the exonic structure of the 5′ ends were considered redundant, and only the longest isoform was retained. Although the high mapping percentage was achieved, we noticed small indels accumulated in 33.6% of the collapsed transcripts (avg. ∼1.6 indels/transcript) (Supplementary Table 1). Small indels were previously reported in 56.2% of the FL transcripts identified from a mouse neural differentiation PacBio dataset (Tardaguila et al., 2018). Previous efforts indicated that correction of indels with matching short reads decreased the number of transcripts harboring indels to 16% but was not satisfactory for open reading frame (ORF) prediction (Tardaguila et al., 2018). Thus, in our study, we used a reference-guided error correction; all collapsed isoforms were mapped back to the genome by SQANTI2, which returned a corrected PacBio reference transcriptome. In a previous study, a hybrid error correction approach using short reads and TAPIS (reference-guided error correction) yielded a 96% mapping percentage compared with 95% using TAPIS suggesting achievement of a high alignment rate without Illumina short reads (Abdel-Ghany et al., 2016).

### Filtration and Characterization of the PacBio Isoforms

Tardaguila et al. (2018) recommended applying quality filters on the PacBio sequencing data to avoid potential technical artifacts due to reverse transcriptase (RT) template switching and off-priming. RT switching is enhanced by RNA secondary structures, which allow RTs to jump without terminating cDNA synthesis leading to gaps that could be interpreted as splicing events. Additionally, the oligo(dT) primer may anneal to non-poly(A) tail in A-rich regions of the template resulting in false cDNA molecules. To investigate the possible intrapriming, the percent of genomic “A’s” in downstream 20 bp from the TTS were calculated. Thus, we adopted various approaches to remove potential technical artifacts from the PacBio transcriptome, including short-read support (Accession # PRJNA389609, PRJNA380337, PRJNA227065, and PRJNA259860), intrapriming, and RT-switching activities (Supplementary Figure 1). Overall, quality filters removed 31,641 transcripts (Supplementary Figure 2). The remaining transcriptome had 76,860 transcripts encoded by 24,729 (95.9%) known genes and 1,068 (4.1%) novel genes when compared with the RefSeq annotation reference (Supplementary File 1). In total, 65,670 ORFs of length ≥ 100 amino acids were predicted (Supplementary File 2). The predicted ORFs were mapped to the Swiss-Prot, TrEMBL, and Pfam protein domain databases (Supplementary Table 2). A total of 62,951 (96%) transcripts had homology with at least one database entity, whereas 49,690 (76%) transcripts had significant matches in the three databases (*E*-value < 10^–5^). Among all collapsed isoforms in our data, there were 2,719 (∼4%) transcripts with predicted ORFs and no matches to any of the protein databases.

Notably, 11,190 transcripts (14.6%) had no ORFs greater than 100 amino acids long, suggesting that they are non-coding transcripts. To confirm the non-coding potential of those transcripts, they were searched against rainbow trout pre-miRNAs (459 records) (Juanchich et al., 2016) and all the miRNA stem-loop sequences (38,589 sequences) available in the miRbase and Rfam (*E*-value ≤ 1e-10). A total of 489 transcripts exhibited homology with 92 miRNA precursors (Supplementary Table 3). There were 10,701 transcripts without homology with miRNA precursors and other non-coding RNA families in Rfam. Those transcripts were processed for lncRNA prediction as we previously described (Al-Tobasei et al., 2016). In total, 4,292 transcripts had a coding score ≤ 1 (Supplementary Tables 4, 5) and, therefore, were considered as lncRNAs. Interestingly, ∼59% of these lncRNAs were non-coding variants of protein-coding transcripts, missing 5′ exons and/or 3′ fragments than their coding transcript counterparts (Supplementary Table 4). Conversion of protein-coding RNA to non-coding RNA has been reported in some bifunctional coding genes, including activating signal cointegrator 1 complex subunit 3 (*ASCC3*) (Williamson et al., 2017), steroid receptor RNA activator 1 (*SRA1*) (Hube et al., 2006), and Protein Phosphatase 1 Nuclear Targeting Subunit (*PNUTS*) (Grelet et al., 2017). For instance, the ASCC3 mRNA switches to a shorter non-coding isoform due to alternative last exon splicing (Williamson et al., 2017). The short non-coding isoform has opposite effects on transcription recovery in response to UV-induced DNA damage (Williamson et al., 2017). *LncRNA–mRNA* hybrid genes need an in-depth investigation to unveil their biological regulatory mechanisms.

**FIGURE 2:**
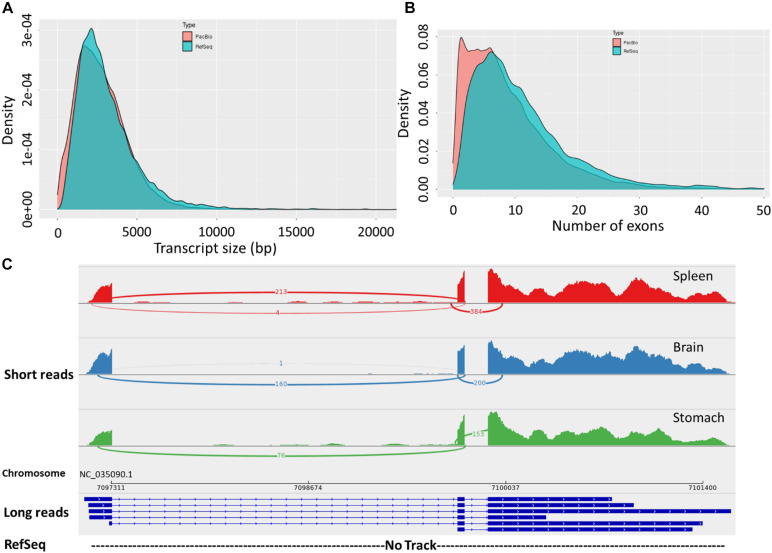
**(A)** Length distribution of FL transcripts obtained from Iso-Seq data compared with RefSeq transcripts. **(B)** Distribution of the number of exons in the long-read sequences and RefSeq transcripts. **(C)** Sashimi plot showing an example of novel transcript isoforms on Omy14, detected by the PacBio Iso-Seq (long-reads track). The bottom (empty) track shows no corresponding annotation for these isoforms in the RefSeq reference. The top three tracks show short reads from three tissues precisely mapped to the exonic structures of the long-read track.

The final corrected transcripts were compared to the RefSeq genome annotation (Release 100; GCF_002163495.1) (Figure 2 and Supplementary Tables 1, 6). There were 32,364 (42.1%) full splice match (FSM) isoforms that perfectly matched reference transcripts at all splice junctions (Supplementary Table 1). About 25.4% of the zebrafish long-read dataset showed an exact match to the RefSeq annotated transcripts (Nudelman et al., 2018). This result suggests the presence of a significant fraction of undiscovered transcriptional diversity in the current RefSeq annotation. Also, 17.4% incomplete splice match (ISM) transcripts were identified as partially matching the reference genome. In zebrafish, 14.8% ISM transcripts lacking 5′– and 3′ end exons were identified (Nudelman et al., 2018). Furthermore, 31,125 (40.5%) novel isoforms were identified in this study. Remarkably, the PacBio isoforms had a fewer average number of exons (avg. 8.8 vs. 11.7 exons) and isoforms per gene compared with the RefSeq transcripts (avg. 3 vs. 4.7 isoforms). We also noticed that novel genes, compared with the RefSeq annotation, tend to have a single multi-exonic (avg. 3.6 exons) isoform per gene. The distribution of isoforms per gene and transcript lengths by structural categories are shown in Supplementary Figures 3a–c.

**FIGURE 3:**
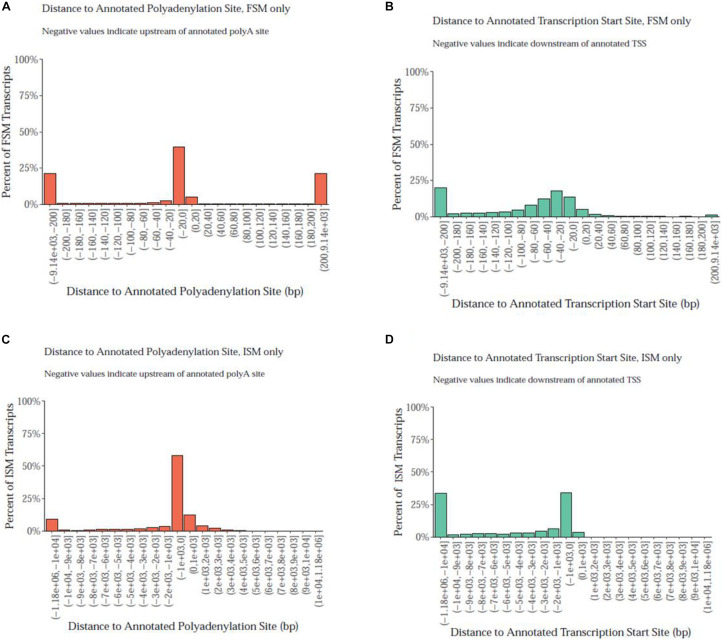
Distance of PacBio transcript ends relative to the reference genome polyadenylation and transcript start sites. **(A)** At the 3′ end, ∼41% of FSM transcripts had an exact or close to complete overlap with the matched reference transcripts. **(B)** At the annotated 5′ end, ∼15% of FSM transcripts showed a complete or close to complete overlap with the matched reference transcripts. **(C)** More than 50% of 3′ ends of ISM sequences fell short within 1 kb upstream the reference annotated 3′ end. **(D)** More than 25% of ISM transcripts had short 5′ ends at 10 kb downstream of the reference 5′ end.

Notably, the PacBio isoforms (2.8 kb) are significantly shorter than the RefSeq transcripts (3.2 kb) on average (*t*-test, *p* = 3.05 × 10^–243^). The distances of 3′ end and 5′ end of FSM and ISM transcripts from the annotated polyadenylation and transcription start sites were calculated, respectively (Figure 3). Within 20 nt upstream of the annotated polyA site, only 41% of FSM transcripts had an exact or close to complete overlap with the 3′ end of matched reference transcripts (Figure 3A). In contrast, ∼15% of FSM transcripts showed a complete or close to complete overlap with the annotated 5′ end (Figure 3B). Additionally, it was obvious that more than 50% of 3′ ends of ISM sequences were falling short within 1 kb upstream of the reference annotated 3′ end (Figure 3C). Most of the ISM transcripts had short 5′ ends, particularly within 1 and 10 kb downstream of the reference 5′ end (Figure 3D). This result agrees with the notion of less control over the completeness of 5′ ends during cDNA library preparation. The imperfect matches between both ends of the PacBio FSM transcripts and reference transcripts may indicate APA/alternative TSS events (Tardaguila et al., 2018). Further investigation using specialized methods in resolving transcript termini is warranted.

### Alternative Splicing and Polyadenylation Modes

**FIGURE 4:**
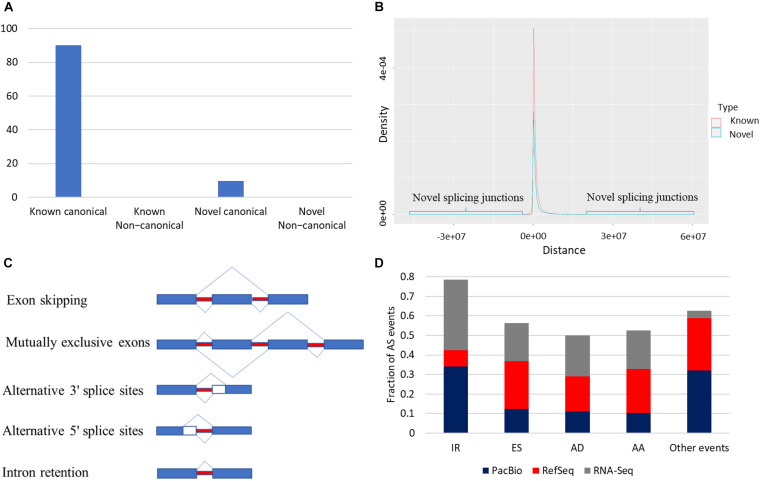
**(A)** Most of the identified splice junctions were from the known canonical category (90.3%). **(B)** Novel splicing junctions tend to be far from the RefSeq transcription start site (TSS) compared with known junctions. **(C)** Types of AS. **(D)** Intron retention was the most frequent AS event in the corrected PacBio transcriptome and RNA-Seq data. In contrast, exon skipping (ES) predominated the RefSeq.

Splice junctions were classified as canonical and non-canonical according to the dinucleotide pairs at the beginning and end of the encompassed intron (Tardaguila et al., 2018) (Supplementary Table 6). Junctions harboring GT-AG, GC-AG, and AT-AC were considered canonical, whereas other possible combinations were non-canonical splicing. Junctions in the reference were described as known junctions; otherwise, they were considered novel junctions. In total, 203,490 splice junctions from the collapsed isoforms were identified. Most of the identified splice junctions were from the known category (90.3%) (Figure 4A). Out of 183,785 known splice junctions, 183,655 (99.9%) were canonical, and only 130 (∼0.1%) were non-canonical. In humans, canonical splice junctions were identified in more than 99.9% of all introns (Cocquet et al., 2006; Parada et al., 2014). Of note, novel junctions were found far from the TSS compared with known junctions (Figure 4B); ∼99.3% of the known canonical junctions were supported by short reads, whereas ∼57% of the novel canonical junctions were validated (Supplementary Figure 4). Notably, less than 1% of the novel non-canonical junctions were supported by short reads (Supplementary Figure 4). Following filtration, 96.7% of the remaining novel non-canonical junctions were supported with short reads. Splice junctions are described in detail in Supplementary Figures 4, 5.

Reconstruction of the rainbow trout transcriptome revealed that 20,431 loci (79.2%) are multi-exonic (avg. ∼9.6 exons). As shown in Figure 4C, AS events were extracted from the annotation file generated from the PacBio dataset (Figure 4D). A total of 33,383 AS events were identified from the PacBio dataset. IR was the most abundant AS event (34.15%) followed by exon skipping (ES) (12.34%) (Figure 4D). On the contrary, in the RefSeq annotation, ES was the most frequent event (24.44%), whereas IR was the least represented one (8.15%) (Figure 4D). Differences may be due to RefSeq annotation being combined from many tissues and different experimental conditions. A recent study reported 16–20% of IR of the genes in mouse and human cortex (Jeffries et al., 2020).

To validate the PacBio findings, the frequency of six types of AS (alternative 3′ splice sites; alternative 5′ splice sites; ES; multiple exon skips, ME; mutually exclusive exons, MX; and IR) was evaluated by RNA-Seq dataset generated from 13 tissues (Accession # PRJNA389609). In agreement with the PacBio data, IR was the most frequent event (36.3%), suggesting the reliability of the findings obtained from PacBio. IR and ES were reported as major AS forms in eukaryotes, with ES higher in animals and IR frequent in all eukaryotes, including plants (Grau-Bove et al., 2018). Our findings improved the transcriptome catalog for rainbow trout.

**FIGURE 5:**
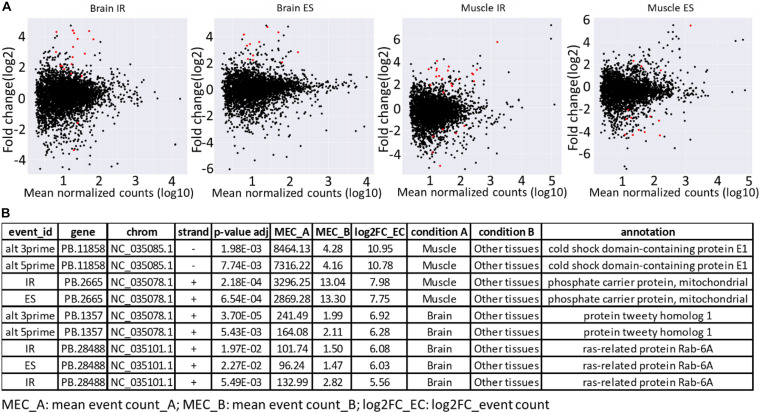
**(A)** MA plots showing major regulated AS forms (IR and ES) in brain and white muscle compared with eight other tissues. The red dots represent the differentially regulated AS forms at adjusted *p* ≤ 0.05. **(B)** Top tissue-specific AS patterns in brain and white muscle.

Furthermore, the availability of a PacBio-improved genome annotation facilitated the identification of differentially regulated AS patterns among tissues. Short-read datasets from nine tissues, collected from two Swanson fish (Berthelot et al., 2014; Salem et al., 2015), were mapped to the Swanson reference genome. A total of 156 differentially regulated events were identified (Supplementary Tables 7, 8). Of them, 33.3% were IR, whereas 21.8% were ES. The splicing event was considered as tissue specific when the event counted in a tissue was at least eight-fold higher than the other tissues (log2 FC > 3; adj. *p*-value < 0.05). A total of 66 splicing events in 44 unique genes were identified as tissue specific (Supplementary Tables 7, 8). Of them, 39.4% were IR, whereas 21.2% were ES. Brain and white muscle had 89% of the tissue-specific splicing events (Supplementary Table 7 and Figure 5A). Similar to our findings, Rodriguez et al. (2020) reported a high abundance of tissue-specific alternative forms in nervous and muscle tissues. A few tissue-specific splicing events were identified from liver, head kidney, and stomach. It is worth mentioning that no differentially regulated/tissue-specific events were identified in spleen, kidney, intestine, and gill when independently compared with other tissues. The top tissue-specific AS patterns in muscle were identified in genes encoding cold shock domain-containing protein E1 (*CSDE1*) and phosphate carrier protein, mitochondrial (*SLC25A3*) (Figure 5B). *CSDE1* is critical for the efficient formation of stress granules (Youn et al., 2018). *SLC25A3* transports inorganic phosphate (Pi) across the mitochondrial membranes, which is necessary for the final step of oxidative phosphorylation. Pathologic variants of the *SLC25A3* have been reported in association with skeletal myopathy phenotype in humans (Mayr et al., 2011; Bhoj et al., 2015). In comparison, the top tissue-specific AS forms in the brain were identified in genes coding for protein tweety homolog 1 (*Ttyh1*) and ras-related protein Rab-6A (*Rab6a*) (Figure 5B). In mammals, the expression of the *Ttyh1* gene is mainly restricted to nervous tissue, where it revealed a role in cell adhesion and as a transmembrane receptor (Matthews et al., 2007). *Rab6a* knockdown led to defects of the cytoskeletal structures in mice (Ma et al., 2016). Tissue-specific alternative forms were previously identified in genes related to cytoskeleton, cell-cell adherens junction, focal adhesion, and structure of muscle fibers (Rodriguez et al., 2020). Further investigation is needed to study the role of tissue-specific AS forms in muscle and brain development.

PacBio sequencing generates FL transcripts containing poly(A) tails, which help to detect APA sites accurately. We searched for possible motifs within 50 nt upstream of the polyadenylation sites. We detected 14 poly(A) signals located within ∼18 nt upstream of the polyadenylation cleavage site (Supplementary Figure 6). The AATAAA (60.6%) and ATTAAA (19.8%) were the most frequent motifs in the PacBio transcriptome (Supplementary Figure 6), suggesting that these motifs are essential for polyadenylation. AATAAA is a well-known conserved poly(A) signal in plants (Feng S. et al., 2019) and animals (Proudfoot and Brownlee, 1976).

### Reconstruction of Coding Regions From the Unmapped/Poorly Mapped Reads

There were 103,193 reads that were unmapped or poorly mapped to the genome and filtered out due to low alignment identity and coverage. The COding GENome reconstruction Tool (Cogent) was used to reconstruct coding regions from the unmapped and poorly mapped reads, generating 10,057 gene families and 8,636 unassigned sequences (Tseng, 2020). All coding bases in isoforms transcribed from a single locus were combined, yielding a reconstructed contig representing each gene family. All reconstructed sequences (*n* = 30,445) (Supplementary Files 3, 4) were employed as a reference to realign the unmapped/poorly mapped reads and make them suitable to be processed through the ToFU pipeline, which filters out reads exhibiting identity less than 0.95 and alignment coverage below 0.99 to each gene family locus. Afterward, redundant isoforms were successfully collapsed into 60,926 FL isoforms (avg. length = 2.9 kb), harboring 41,414 ORFs (Supplementary File 5 and Supplementary Figure 7). Collapsed isoforms were annotated as shown in Supplementary Tables 9–11. Remarkably, when all collapsed reconstructed transcripts were mapped to the Swanson genome sequence, only 388 (0.64%) transcripts were mappable at identity ≥ 0.95 and coverage ≥ 0.99. In contrast, when the 60,926 reads were mapped to the newly released genome sequence of rainbow trout Arlee strain [USDA OmykA_1.1 assembly (GCF_013265735.2)], 35,218 (∼58%) transcripts were mappable, suggesting the reliability of the Cogent reconstructing the coding sequences and perhaps a necessity to improve the current version of the Swanson strain genome reference of this study. It is worth mentioning that the contiguity of the Arlee genome assembly has recently been improved using long reads. Furthermore, the Bionano optical mapping and Hi-C proximity ligation sequence data were used to join the Arlee contigs into scaffolds, which were then anchored to and ordered on chromosomes using genetic linkage information. The Swanson genome assembly has 139,799 unplaced scaffolds compared with 939 scaffolds in the Arlee assembly (Gao et al., 2021).

**FIGURE 6:**
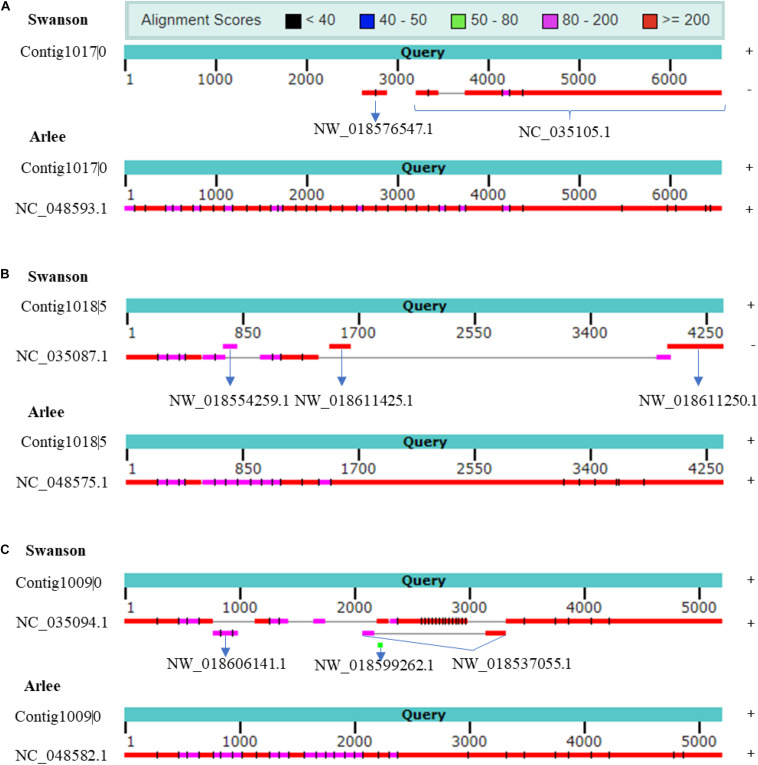
**(A)** Cogent contig shows chromosome NC_035105.1 (Omy29) missing ∼3.2 kb. Cogent contig shows that the genomic scaffold NW_018576547.1 is placed on Omy29. The reconstructed contig aligns to chromosome Y on Arlee assembly, which provides evidence for the accuracy of Cogent reconstruction. **(B)** Cogent contig shows chromosome NC_035087.1 (Omy11) missing ∼2.5 kb. Cogent reconstructed one contig to which three scaffolds were mapped in this order, namely, “NW_018554259.1,” “NW_018611425.1,” and “NW_018611250.1” and validated by Arlee assembly. **(C)** The reconstruction reveals several discrete misassemblies on Omy18 (NC_035094.1) and anchors three scaffolds to the chromosome. Arlee assembly was used to validate the reconstruction. Strand orientations are provided on the right side of the figure. Cogent contigs and Arlee loci have the same strand orientations.

rnaQUAST 1.2.0^1^ was used to further assess the PacBio transcriptome quality compared with Swanson RefSeq (Bushmanova et al., 2016). The collapsed isoforms were mapped to the Swanson trout reference genome using GMAP and BLAT to match the alignments to the reference coordinates (Supplementary Tables 12, 13). Based on the common alignment output, a total of 1,479 collapsed transcripts showed no significant alignment with the Swanson trout genome; of these, 346 transcripts (23%) had significant hits with the Arlee strain (identity ≥ 0.95 and coverage ≥ 0.99), suggesting those transcripts are missing in the Swanson RefSeq annotation; 8,348 unannotated transcripts did not match any reference transcripts. The mapping revealed 15,120 misassembled transcripts (mapped to a different chromosome, strand, reverse order, etc.). To prove that the misassembled transcripts are not due to a high error rate in the PacBio sequencing, we mapped the ∼15K misassembled transcripts to the Arlee and Atlantic salmon genome sequences. A total of 9,935 (∼66%) and 1,209 (∼8%) transcripts matched the Arlee and Atlantic salmon genomes at identity ≥ 0.95 and coverage ≥ 0.99. For instance, Iso-Seq identified seven isoforms; Cogent resolved it to one contig. Mapping the contig back to NC_035105.1 (Omy29) showed a misassembly where the strand orientation is opposite, and Omy29 is missing the first ∼3.2 kb of the contig (Figure 6A). The Arlee assembly provided evidence for the presence of the 3.2 kb on the Y chromosome (Figure 6A) and, in turn, the accuracy of our reconstructed contig. Similarly, the reconstruction yielded a contig mapped to Omy11, which lacks ∼2.5 kb (Figure 6B). The contig also maps to three unplaced genomic scaffolds: “NW_018554259.1,” “NW_018611425.1,” and “NW_018611250.1” (Figure 6B). Also, Iso-Seq identified six transcripts that Cogent reconstructs into a single contig. The reconstructed Cogent contig, mapped to Omy18 and three scaffolds, showed that scaffold order should be “NW_018606141.1” followed by “NW_018537055.1” and “NW_018599262.1” (Figure 6C). Overall, Arlee assembly provided evidence for the accuracy of contig reconstruction, suggesting the necessity to refine the gene models in the current Swanson genome assembly.

The completeness of the PacBio transcriptome was assessed using Benchmarking Universal Single-Copy Orthologs (BUSCO) (Seppey et al., 2019). BUSCO v5.1.2 checked for single and duplicate orthologs for members of the Actinopterygii lineage. A total of 3,640 BUSCO groups were searched to assess the transcriptome completion. Overall, 89% annotation completeness was achieved. BUSCO alignments revealed 8,679 well-mapped FL transcripts and 2,564 FL unmapped/poorly mapped collapsed transcripts that have hits to 2,662 and 1,185 orthologs, respectively. Remarkably, the unmapped and poorly mapped transcripts had hits to 564 orthologs with no matches in the well-mapped transcripts. Our results showed that the characterization of the rainbow trout transcriptome is close to complete and that sequencing more tissues from different biological conditions may help identify more FL transcripts to complete the genome annotation.

### Alternative Splicing, Polyadenylation, and TSS Associated With Economically Important Phenotypes

AS and APA are interesting complexity aspects of the eukaryote transcriptome. The mechanism of AS and APA generates more transcripts per gene locus and, thus, expands the proteome diversity. Previous studies showed that the posttranscriptional mechanisms play important roles in immune responses (Martinez and Lynch, 2013), muscular atrophy (Lorson et al., 1999), cancer (Hube et al., 2006), and neurological disorders (Zhou et al., 2008). Therefore, we used DEXSeq to profile differential exon usage (DEU) in rainbow trout across different biological conditions using publicly available data (see “Materials and Methods” section) to identify AS and APA associated with the studied phenotypes. Change in relative exon usage could be due to (1) a change in the rate of exon splicing (i.e., AS), (2) a change in usage of alternative TSS, or (3) a change in usage of APA sites.

#### Fish Growth and Muscle Accretion

To identify AS and APA events contributing to fish growth and muscle accretion, RNA-Seq data previously generated from fish families exhibiting extreme whole-body weight (WBW) and muscle yield phenotypes (Ali et al., 2018) were mapped to the rainbow trout genome using TopHat2. DEU analysis revealed two exons differentially spliced in fish families showing divergent WBW phenotypes (Supplementary Table 14). The spliced transcripts are coding for the negative elongation factor C/D (*NELFCD*) and titin genes. The differentially used exon (DUE) in *NELFCD* (NC_035093.1 :41691708-41692396) was upregulated in fish families with low WBW (Supplementary Figure 8). Knockdown of *NELFCD* suppressed cancer cell proliferation *in vitro* (Song et al., 2018). Conversely, the DUE in titin (exon9) was upregulated in fish families with high WBW. Titin guides the assembly of myofibrils from premyofibrils. In zebrafish, knockout of titin from two titin homologs developed exon-dependent phenotypes of variable severity, including susceptibility to biomechanical stresses and degeneration during development explained by the exon usage hypothesis (Shih et al., 2016). Additionally, a single exon (NC_035087.1:57851656-57851747) was significantly DU and upregulated in fish families showing high muscle yield (Supplementary Figure 8 and Supplementary Table 14). This exon is in a novel isoform coding for THO complex subunit 5 homolog (*THOC5*). *THOC5* is an essential element for normal proliferation and differentiation processes (reviewed in Tran et al., 2014). Depletion of *THOC5* in the embryonic fibroblasts inhibited cell growth (Guria et al., 2011). It is noteworthy that all identified DUEs were in the perfectly mapped transcripts.

**FIGURE 7:**
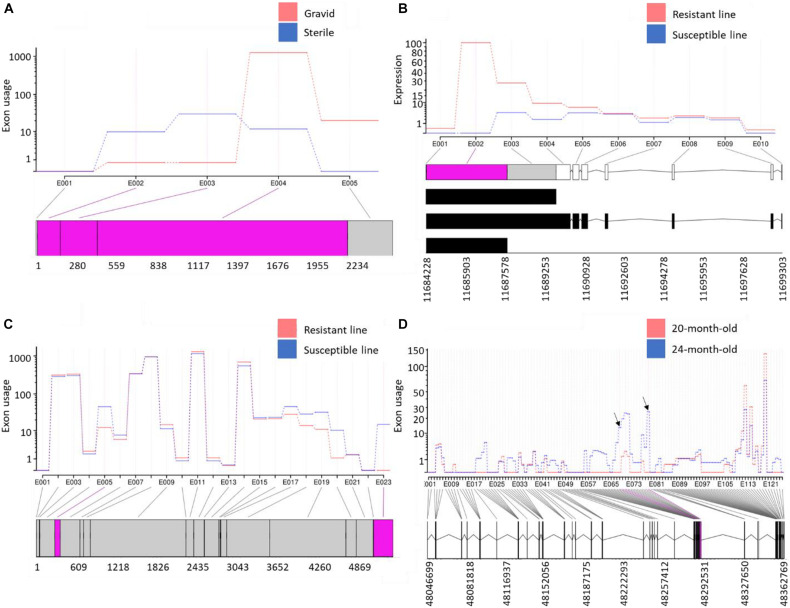
**(A)** Variable exonic features of isoforms transcribed from a gene encoding MHC class I heavy chain showed opposite usage patterns in atrophying muscle during sexual maturation; **(B)** the first exon in a gene encoding *GVIN1* revealed exceptional downregulation in fish from the susceptible genetic line for BCWD; **(C)** a non-coding transcript was alternatively polyadenylated in fish showing divergent resistance to BCWD; and **(D)** exceptional upregulation of two exons in novel isoforms, encoding Ig mu chain C region membrane-bound form, in anadromous female smolts (24-month-old) compared with presmolts (20-month-old). Differentially used exonic features are shown in pink at the bottom of each panel.

To identify AS and APA events involved in muscle atrophy associated with sexual maturation, RNA-Seq data previously generated from gravid and sterile rainbow trout were used (Paneru et al., 2018). A total of 747 DUEs (adj. *p*-value < 0.05) were identified (Supplementary Table 14). The eukaryotic translation initiation factor 4E binding protein 2 (*EIF4EBP2*) had the most significant DUE (exon3; log2 FC = 4.4; adj. *p*-value = 4.44E-47) in the sterile fish relative to gravid fish. *EIF4EBP2* is known to inhibit protein synthesis, and the mTOR signaling pathway inactivates it to stimulate cell growth and metabolic process (Ding et al., 2018). Since this exon is highly used in sterile fish, we speculate that this exon is likely inactivating the *EIF4EBP2*. Conversely, mucosa-associated lymphoid tissue lymphoma translocation protein 1-like (*MALT1*) and four other genes had exons totally absent in the sterile fish. *MALT1* is a signaling component with protease functions (Coornaert et al., 2008). A total of 258 exons in the reconstructed poorly mapped/unmapped transcripts were DU (Supplementary Table 15). Of them, MHC class I heavy chain (PB.5976) (Figure 7A) and protein-tyrosine kinase 2-beta (PB.17301) were at the top of the list. Gene enrichment analysis revealed that the isoforms harboring DUE are significantly enriched in the ribosome KEGG pathway and have GO terms belonging to translation (Supplementary Table 14).

#### Disease Resistance

*Flavobacterium psychrophilum*, the causative agent of BCWD, causes worldwide economic losses to the aquaculture industry (Nematollahi et al., 2003). Resistance to BCWD was demonstrated to be a moderately heritable trait that responds to selection (Silverstein et al., 2009; Leeds et al., 2010). Selective breeding programs have the potential to improve heritable phenotypes through existing genetic variation among individual animals or families (Ali et al., 2020). To gain insights into the molecular mechanism associated with resistance BCWD, RNA-Seq datasets previously generated from two genetic lines exhibiting contrasting resistance to BCWD (Marancik et al., 2014) were used for exon usage analysis (Supplementary Tables 14, 15). On day 1 post-infection, 77 exons were DU in the resistant and susceptible genetic lines. Of them, the first exon in a gene encoding interferon-induced very large GTPase 1 (*GVIN1*) was upregulated in the resistant line (log2 FC = 18.0). *GVIN1* was differentially expressed among survivors of three carp clones following herpesvirus (*Ca*HV) challenge (Lu et al., 2019). DEU analysis of all transcriptomic datasets from resistant and susceptible genetic lines showed that, regardless of the infectious status and days of infection, the *GVIN1* exon (Figure 7B) was completely absent in the susceptible line (log2 FC = 20.9; Supplementary Table 14). To further validate these data, the *GVIN1* exon was amplified by qPCR; the exon expression level in the resistant line exceeded that from the susceptible line by about 25-fold (Supplementary Figure 8). Transcript abundance analysis revealed that one of the three transcripts harboring the *GVIN1* exon was the most upregulated transcript in the resistant line (log2 FC = 2.5) compared with the susceptible line (Supplementary Table 17). The *GVIN1* DUE encodes 670 amino acids representing 36% of the whole ORF (1,836 amino acids long). These results suggest a role for *GVIN1* in disease resistance to BCWD.

**FIGURE 8:**
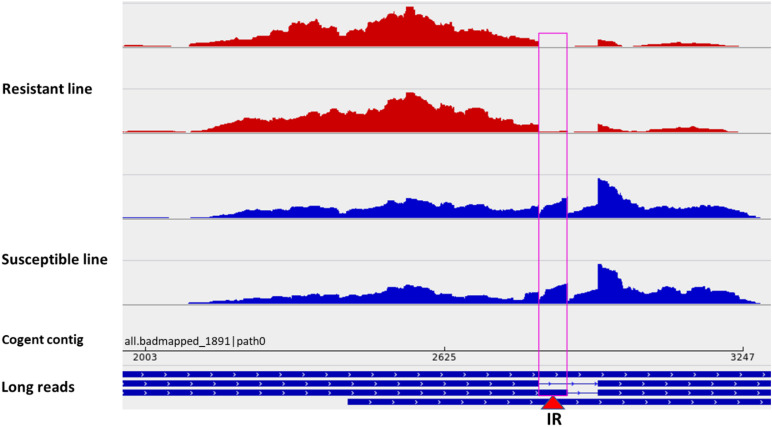
A small/partial intron retention event in a transcript isoform coding for pentraxin-related protein PTX3 (red triangle). Two short-read datasets from a BCWD-resistant genetic line (red panels) showing no intron retention and two sets from a susceptible genetic line showing intron retention (blue panels).

Among all datasets from resistant and susceptible genetic lines (eight RNA-Seq dataset/genetic line), 238 DUEs were identified in the reconstructed unmapped/poorly mapped contigs (Supplementary Table 15). For instance, exon 3 in three isoforms coding for dystrophin was completely absent in resistant fish and showed log2 FC = –13.6 when compared with fish from the susceptible line (Supplementary Figure 8). In addition, an exon in a non-coding transcript (Figure 7C) and an exon in a transcript encoding microfibril-associated glycoprotein 4 were alternatively polyadenylated in fish showing divergent resistance to BCWD. In *Oreochromis niloticus*, a microfibril-associated glycoprotein 4 has demonstrated agglutination and opsonization capability to bacteria (Wu et al., 2020). A small/partial IR event was detected in a transcript coding for pentraxin-related protein PTX3 (Figure 8). The latter is a mediator of innate resistance to bacterial pathogens (Doni et al., 2019).

Our results indicate substantial genetic variation among fish from the resistant and susceptible lines explained by DUEs.

#### Fish Migration

To identify the molecular mechanism driving fish migration, we sought to compare the brain transcriptome at the time of smoltification (i.e., the physiological transition into seagoing forms) to an early time point during the second year of development in both male and female anadromous fish. For this purpose, an RNA-Seq dataset was obtained from Hale et al. (2016). A total of 533 and 349 DUEs belonging to the well-mapped and reconstructed reads were identified in anadromous female fish during smoltification (24 months) compared with 20-month-old presmolt fish (Supplementary Tables 14, 15). The DUEs were not identified in the resident rainbow trout population simultaneously (Supplementary Table 14), suggesting a potential role in fish migration. The list of DUE included downregulation of the first exon of an isoform encoding glycogen phosphorylase B (log2 FC = –16.9; Supplementary Tables 14, 15). During migration, muscle proteins in salmon act as a fuel and a carbon skeleton source to maintain the hepatic glycogen levels (Halver and Hardy, 2002). Hepatic glycogen content in Atlantic salmon after seawater entry became much lower (Plisetskaya et al., 1994). This may explain the complete absence of the glycogen phosphorylase exon at the 24-month-old fish. Also, the upregulation of two exons in a gene coding for E3 ubiquitin-protein ligase RNF115, which is involved in protein ubiquitination, may provide evidence of the role of proteins as a fuel source during migration. Our analysis revealed enrichment of the DUE-harboring isoforms in carbohydrate metabolism and ribosome KEGG pathways. Up on smoltification (24 months), the usage of two small exons belonging to the gamma-aminobutyric acid receptor-associated protein (*GABARAP*) and serine/threonine-protein phosphatase 2A catalytic subunit alpha isoform (*PPP2CA*) dramatically decreased. *GABARAP* has a role in increasing the activity of the major inhibitory neurotransmitter (GABA), which is associated with behavioral traits in mice and Atlantic salmon (Thornqvist et al., 2015). Hypomethylated cytosines associated with *PPP2CA* were previously identified in 20-month-old fish relative to 8-month-old fish (Gavery et al., 2019). We noticed exceptional upregulation of two exons in novel isoforms expressed from a gene encoding Ig mu chain C region membrane-bound form (Figure 7D). Changes in immune response in migrating salmon were previously reported not to be due to infection but rather to the life history of salmon (Dolan et al., 2016). Upregulated exon was identified in a transcript encoding unconventional myosin-VIIa, which is required for sensory perception of the light stimulus (Ahmed et al., 2001) and sound (Weil et al., 1995). Migratory salmon rely on the sensory system (Farrell, 2011). Several other DUEs were identified in transcripts encoding proteins with a role in maintaining the nervous system such as Aladin (Tullio-Pelet et al., 2000); Na/K ATPase alpha subunit isoform 1b (Peng et al., 1997; Edwards et al., 2013); glycerophosphodiester phosphodiesterase 1 (Yanaka, 2007); protein-arginine deiminase type-2 (Asaga and Ishigami, 2007); lysyl oxidase homolog 2A (Du and Zhu, 2018); glutamate receptor ionotropic, kainate 2 (*GRIK2*) (Martin et al., 2007); and ST8 alpha-*N*-acetyl-neuraminide alpha-2,8-sialyltransferase 1 (Husain et al., 2008).

A total of 1,163 and 164 DUEs in the well-mapped and reconstructed reads, respectively, were identified among 20– and 24-month-old anadromous males (Supplementary Tables 14, 15). Functional annotation analysis showed that the DUE-harboring transcripts are enriched in brain development, axon extension, response to activity, ATP binding, and tricarboxylic acid cycle. Remarkably, only 10 DUEs were common between male and female smolts. The list included protocadherin alpha-C2 (*PCDHAC2*) and *GRIK2*. *PCDHAC2* is involved in establishing and maintaining complex networks of neuronal connections in the brain (Wu and Maniatis, 1999), and *GRIK2* has GO terms related to the detection of cold stimulus involved in thermoception. Atlantic salmon smolts start their migration at a water temperature of 5°C and reach a peak of migration at water temperature > 8°C (Whalen et al., 1999). The current study showed sex-biased exon usage and suggests a role for AS in regulating the developmental plasticity in anadromous fish toward smoltification.

#### Response to Stress

**FIGURE 9:**
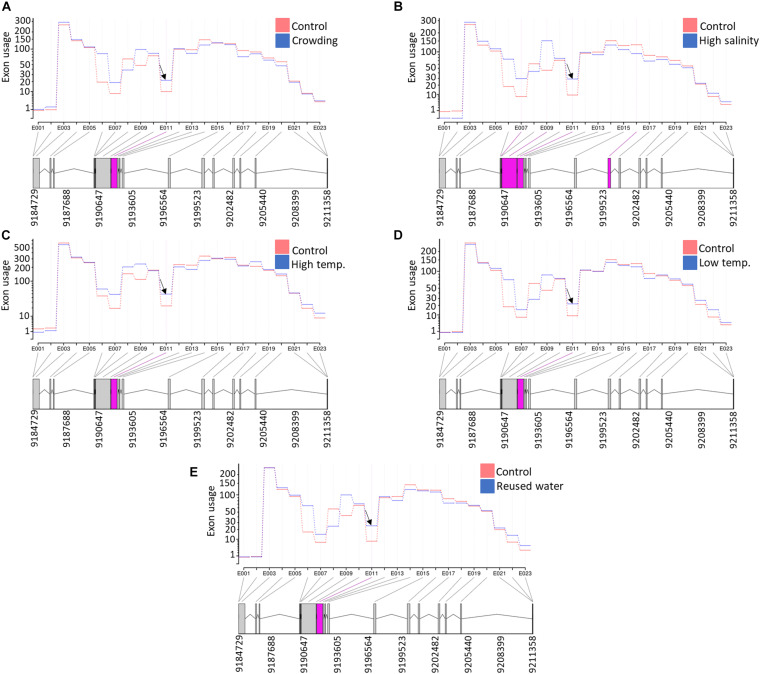
A single exon (NC_035086.1:9191793-9192329) in an isoform encoding prolyl 4-hydroxylase subunit alpha-2 (*P4HA2*) was differentially used under all five studied stress conditions [**(A)** crowding, **(B)** high salinity, **(C)** high temperature, **(D)** low temperature, and **(E)** reused water]. Differentially used exonic features are shown in pink at the bottom of each panel.

Under intensive rearing conditions, fish experience diverse stressors, which negatively affect fish health, growth, and filet quality. Understanding the molecular mechanisms underlying stress responses will help to develop strategies that target improving animal welfare and aquaculture industry profitability. Therefore, we investigated DUEs in rainbow trout fish under five different stress conditions. For this purpose, RNA-Seq datasets were downloaded from the NCBI SRA (PRJNA312486). A total of 665, 37, 286, 554, and 124 DUEs were identified in fish exposed to high salinity, high temperature, low temperature, reused water, and crowding, respectively (Supplementary Table 14). Under all five stress conditions, there was a single common DUE (NC_035086.1:9191793-9192329) belonging to transcript isoforms encoding prolyl 4-hydroxylase subunit alpha-2 (*P4HA2*) (Figure 9). Prolyl hydroxylation is a posttranslational modification to modulate protein folding and stability (Xiong et al., 2018). Prolyl 4-hydroxylase requires ascorbate to catalyze hydroxylation of proline residues in newly synthesized collagen chains to form 4-hydroxyproline. The hydroxylated residues stabilize the collagen triple helices under different physiological conditions (Pihlajaniemi et al., 1991). For instance, it was reported that stressed animals have a low concentration of ascorbic acid, which is not sufficient for collagen hydroxylation. This abnormal collagen affects the basement membrane structure of epithelial layers, causing skin lesions and blood vessel fragility (Pandey, 2007).

Three DUEs were identified in response to at least four stressors. Of them, delta-1-pyrroline-5-carboxylate synthase (*P5CSA*) and heat shock protein HSP 90-alpha (*HSP90α*) had an upregulated DUE in response to high salinity, low temperature, reused water, and crowding. In plants, *P5CSA* is induced in response to salt stress (Yoshiba et al., 1995) and water deprivation (Sharma et al., 2011). Pollutant-exposed fish hepatocytes induced *HSP90α*, which enabled the hepatocytes to become tolerant to oxidative stress (Padmini and Usha Rani, 2011). Conversely, two novel antisense transcripts had a downregulated DUE in response to high temperature, low temperature, reused water, and crowding.

A total of 24 DUEs were identified in response to at least three stressors. For instance, six exons were DU in response to high salinity, reused water, and crowding such as angiomotin, adenylate cyclase type 2, and lysine-specific histone demethylase 1A. Salt adaptation in teleost fish modulates the adenylate cyclase activity (Guibbolini and Lahlou, 1987). Acute stress in mouse models was regulated by the lysine-specific demethylase 1 (Longaretti et al., 2020). Fish differentially used three common exons when they were subjected to extreme temperatures. Two of them were belonging to transcripts encoding myozenin-2 and *P4HA2*, and a single DUE belonged to two non-coding antisense transcript isoforms. Transcripts undergoing posttranscriptional events in response to stress are enriched/involved in glycolysis and protein processing in the endoplasmic reticulum (Supplementary Table 16). Stress levels are often assessed according to plasma glucose and lactate levels (Arends et al., 1999; Acerete et al., 2004). Endoplasmic reticulum stress in the hepatopancreas of white shrimp was reported in response to low temperature (Fan et al., 2016). Our findings provide a basis for further investigation of molecular response to stress in rainbow trout, leading to better breeding practices to improve aquaculture production efficiency.

## Conclusion

Iso-Seq data were used to construct a high-confidence FL transcriptome for rainbow trout. The study identified ∼76K FL transcripts that are well-mapped to the current Swanson reference genome and contain ∼65K ORFs longer than 100 amino acids. We identified 1,068 (4.1%) novel gene loci not previously annotated in the RefSeq reference. Additionally, ∼60K FL isoforms that were either poorly mapped or unmapped (∼1.4K transcripts) to the current genome were reconstructed into 30,445 Cogent contigs. Unlike the RefSeq annotation, PacBio and RNA-Seq data revealed that IR is the most frequent AS event in the rainbow trout. The PacBio-improved transcriptome was used to identify AS and isoform expression associated with fish growth and muscle accretion, disease resistance, migration, and stress response. The improved transcriptome provides an avenue for future genetics and genomics studies to enhance aquaculture production efficiency.

## Materials and Methods

### Production of Doubled Haploid Fish

Fish from the Swanson clonal line were obtained from the Washington State University (WSU) trout hatchery. Fish were produced as previously described (Scheerer et al., 1986; Young et al., 1996; Robison et al., 1999). Androgenesis was used to produce first-generation homozygous fish where eggs were gamma-irradiated before fertilization (Young et al., 1996; Robison et al., 1999). Sperms were collected from sexually matured homozygous males to perform another cycle of androgenesis producing homozygous clones (Scheerer et al., 1986). Three fish were dissected to collect tissues for Iso-Seq. Tissues included white muscle, red muscle, kidney, head kidney, spleen, stomach, gill, testis, heart, bone, skin, brain, liver, and intestine. Also, fertilized eggs at different developmental stages were collected.

### Library Preparation and Sequencing

RNA was isolated from frozen tissues using TRIzol reagent (Life Technologies, Carlsbad, CA, United States) according to the guidelines of the manufacturer. RNA integrity was checked using Bioanalyzer (Agilent, Santa Clara, CA, United States^2^). RNA samples with RIN > 9 were used for Iso-Seq library preparation. The Clontech SMARTer PCR cDNA Synthesis Kit was used for first-strand cDNA synthesis according to PacBio instructions.

Following PCR cycle optimization, a large-scale PCR was performed to generate double-stranded cDNA for SMRTbell library construction. AMPure PB Bead Purification of large-scale PCR products was performed and exonuclease was used to remove failed ligation products. AMPure PB beads were used to purify SMRTbell templates twice. The sequencing libraries were prepared by annealing a sequencing primer and binding a polymerase to SMRTbell templates. In total, 19 SMRT cells were sequenced.

### Iso-Seq Analysis Pipeline

The pipeline included three initial steps: generation of CCS subreads, classification of FL reads, and clustering of FLnc reads. Polished CCS subreads were generated, using *CCS* v4.0.0, from the subreads bam files with a minimum quality of 0.9 (–min-rq 0.9). The default minimum number of FL subreads (*n* = 3) required to generate CCS for a zero-mode waveguide (ZMW) was used. FL transcripts were determined when the sequences had the poly(A) and the 5′ and 3′ cDNA primers. *Lima* v1.10.0 and *isoseq3 refine* v3.2.2 were used to remove the primers and poly(A) tails, respectively. The clustering algorithm ICE was used to obtain high-quality FL consensus sequences. The consensus transcripts were mapped to the Swanson rainbow trout reference genome (Pearse et al., 2020) using minimap2-2.17 (r941) (-ax splice -uf –secondary = no –C5 –O6,24 –B4) (Li, 2018). SAM files were sorted and used to collapse redundant isoforms using Cupcake v9.1.1^3^. Unmapped and poorly mapped isoforms were used as input to Cogent v6.0.0^4^ to reconstruct the coding genome. The reconstructed contigs were used as a fake genome to process and collapse the unmapped and poorly mapped reads through the ToFU pipeline.

### Transcriptome Characterization

SQANTI2 v6.0.0 was used to characterize and curate the long-read transcriptome (Tardaguila et al., 2018). The Swanson rainbow trout reference genome sequence [Omyk_1.0 (GCF_002163495.1)], annotation file (GTF), and quantification data were used as input to SQANTI2 to characterize/classify the collapsed isoforms and assess the quality of the sequencing data and the preprocessing ToFU pipeline (Gordon et al., 2015). A reference-guided error correction was implemented. Transcripts were classified into eight structural categories. Transcripts having splice junctions in a complete match with the reference transcripts were labeled as “FSM,” whereas transcripts with partial consecutive matches with the annotated transcripts were labeled as “ISM.” Novel isoforms of known genes were classified into Novel in Catalog “existing in the RefSeq annotation” (NIC) if containing a combination of annotated donor/acceptor sites or into Novel Not in Catalog (NNC) if at least containing one unannotated donor or acceptor site. In addition, “Genic Genomic” isoforms partially overlap with exons/introns of an annotated gene, whereas “Fusion” transcripts span two annotated loci. Transcripts in novel genes, compared with the RefSeq annotation, were classified as “intergenic” if existing outside the body of known genes, “Genic Intron” if completely contained within a known intron, and “Antisense” if overlapping the complementary strand of a known transcript. Potential artifacts were removed using SQANTI machine learning classifier. Transcripts flagged as intrapriming and RT-switching candidates were filtered out. The GeneMarkS-T (GMST) algorithm was implemented to predict ORFs from the corrected transcripts (Tang et al., 2015). Predicted ORFs were mapped to the Pfam protein domain database, Swiss-Prot, and TrEMBL database. The Database for Annotation, Visualization and Integrated Discovery (DAVID) v6.8 was used for gene enrichment analysis (FDR < 0.05) (Huang da et al., 2009).

AStalavista^5^ was used with the raw annotation file generated from the Iso-Seq data to identify and classify AS events. SplAdder (Kahles et al., 2016) was used to identify AS events in rainbow trout tissues using bam files generated from the RNA-Seq datasets (Accession # PRJNA389609 and PRJEB4450) mapped to the trout genome. The frequencies of the six AS events (IR, ES, MX, ME, alternative 3′ splice sites, and alternative 5′ splice sites) and significant quantitative differences among tissues were determined from the SplAdder output files.

### Non-coding RNA

Transcripts lacking ORFs or harboring ORFs less than 100 amino acids long were considered as potential non-coding transcripts. Those transcripts were aligned to all miRNA stem-loop sequences in miRbase^6^ (release 22.1) and trout miRNA precursors (Juanchich et al., 2016) to identify pre-miRNAs. Also, putative non-coding transcripts were aligned to Rfam database to identify miRNAs and other non-coding classes. The remaining transcripts that did not match miRbase, trout miRNA precursors, and Rfam and longer than 200 bp were assessed for coding potential using CPC (CPC score ≤ 1) (Kong et al., 2007) and CPC2 web servers (Kang et al., 2017). Transcripts that were evaluated as non-coding were considered as putative lncRNA transcripts. Finally, these transcripts were aligned to the previous lncRNA assembly from rainbow trout (Al-Tobasei et al., 2016) using BLASTn (*E*-value 1e-5).

### Differential Exon Usage Analysis

FastQC v0.11.9 was used to check the quality of the RNA-Seq datasets generated from rainbow trout fish under different biological conditions. Low-quality sequences were trimmed/removed using Trimmomatic v0.36 (Bolger et al., 2014). High-quality reads were mapped to the reference genome sequence by TopHat2 (Kim et al., 2013) with the default parameters.

DEXSeq package (v1.34.1) (Anders et al., 2012) was used to infer the DEU in the RNA-Seq datasets (Liu et al., 2014; Marancik et al., 2014; Hale et al., 2016; Ali et al., 2018; Paneru et al., 2018). DEXSeq counts the number of reads mapped to each exon (or part of an exon) in all samples. To infer changes in the relative exon usage, DEXSeq considers the change in the ratio of the number of reads mapped to an exon to read counts mapped to other exons of the same gene across conditions. DEXSeq uses two python scripts to prepare the GFF file and count the mapped reads. The first script, *dexseq_prepare_annotation.py*, was used to convert the GTF file with gene models into a gff file with collapsed exon counting bins. The second python script, *dexseq_count.py*, uses the sorted BAM/SAM alignment files to count the number of overlapping reads with each exon counting bin defined in the prepared GFF file. Default parameters were implemented in the DEXSeq analyses.

### RT-PCR Validation of PacBio Isoforms and DEU

Reverse transcription (RT)-PCR was carried out to validate the long-read isoforms and to quantify exon usage as previously described (Ali et al., 2018). Primers used for RT-PCR analysis were designed using Primer3. First-strand cDNAs were synthesized using a Verso cDNA Synthesis Kit (Thermo Scientific, Hudson, NH, United States) following the instructions of the manufacturer. Each qPCR reaction contained a template (100 ng/μl), forward and reverse primers (10 μM working solution), and SYBR Green master mix (Bio-Rad, Hercules, CA, United States). Nuclease-free water was added to each reaction to achieve a final reaction volume of 10 μl. Quantification was performed in triplicates. *β-Actin* gene was used as an internal standard for normalization of expression. The PCR for all reactions started with 95°C for 30 s followed by 40 cycles. Each cycle lasted 15 s at 95°C, 30 s at the appropriate annealing temperature for each primer, and 30 s at 60°C. The expression was quantified using the delta delta Ct (ΔΔCt) method.

## Data Availability Statement

All the raw sequence data for this study were submitted to the NCBI BioProject at https://www.ncbi.nlm.nih.gov/bioproject/PRJNA389609.

## Ethics Statement

The Washington State University Institutional Animal Care and Use Committee reviewed and approved the animal study under protocol #02456.

## Author Contributions

MS conceived and designed the experiments. AA, GT, and MS performed the experiments. AA and MS analyzed the data and wrote the manuscript. GT contributed reagents, materials, and analysis tools. All authors contributed to the article and approved the submitted version.

## Conflict of Interest

The authors declare that the research was conducted in the absence of any commercial or financial relationships that could be construed as a potential conflict of interest.

## Footnotes

1 https://de.cyverse.org/

2 https://www.agilent.com/

3 https://github.com/Magdoll/cDNA_Cupcake

4 https://github.com/Magdoll/Cogent

5 http://astalavista.sammeth.net/

6 http://www.mirbase.org/

## Supplementary Material

Supplementary Figure 1 RT-switching and intra-priming.

Supplementary Figure 2 Isoform distribution across structural categories before and after filtration.

Supplementary Figure 3 Comparison between novel and annotated genes & transcript length across structural categories.

Supplementary Figure 4 Distribution and characteristics of splice junctions.

Supplementary Figure 5 Characterization of splice junctions in the corrected transcriptome generated from PacBio dataset.

Supplementary Figure 6 Location, distribution, and frequency of polyadenylation cleavage site.

Supplementary Figure 7 Characterization of unmapped/poorly-mapped collapsed transcripts.

Supplementary Figure 8 Validation of a subset of differentially used exons by qPCR.

Supplementary Table 1 Classification and annotation of all well-mapped PacBio transcripts.

Supplementary Table 2 Hits of PacBio ORF (predicted from well-mapped transcripts) with three protein databases (Pfam, TrEMBL, and Swiss-Prot).

Supplementary Table 3 PacBio well-mapped transcripts exhibiting homology with rainbow trout pre-miRNAs, miRNA stem-loop sequences available in the miRbase, and Rfam.

Supplementary Table 4 Long non-coding PacBio well-mapped transcripts and their matches with our previous lncRNA assembly.

Supplementary Table 5 Conservation of long non-coding PacBio well-mapped transcripts.

Supplementary Table 6 genomic position and classification of splice junctions in PacBio well-mapped transcripts.

Supplementary Table 7 Differentially regulated AS forms among rainbow trout tissues.

Supplementary Table 8 Genomic coordinates of differentially regulated AS events among tissues (gff-version 3).

Supplementary Table 9 Hits of PacBio ORF (predicted from unmapped and poorly mapped transcripts) with three protein databases (Pfam, TrEMBL, and Swiss-Prot).

Supplementary Table 10 Coding potential of lncRNAs identified from unmapped/poorly-mapped transcripts.

Supplementary Table 11 Unmapped/poorly mapped non-coding transcripts exhibiting homology with miRNA precursors.

Supplementary Table 12 Statistics of rnaQUAST to assess the PacBio transcriptome quality compared to Swanson RefSeq.

Supplementary Table 13 Unaligned, misassembled, and unannotated PacBio transcripts in the Swanson RefSeq genome.

Supplementary Table 14 Differentially used exonic feature of well-mapped transcripts under different biological conditions.

Supplementary Table 15 Differentially used exonic feature of unmapped/poorly-mapped transcripts under different biological conditions.

Supplementary Table 16 Gene enrichment analysis of transcripts harboring differentially used exons.

Supplementary Table 17 Differentially expressed transcripts between fish from resistant and susceptible genetic lines for BCWD.

Supplementary File 1 Genomic coordinates of all well-mapped PacBio transcripts (gtf file).

Supplementary File 2 ∼65K predicted ORFs from well-mapped PacBio transcripts (FASTA format).

Supplementary File 3 ∼30K Cogent reconstructed contigs (FASTA format).

Supplementary File 4 Annotation file for all unmapped/poorly mapped transcripts (gff file).

Supplementary File 5 41K predicted ORFs from unmapped/poorly mapped transcripts (FASTA format).
